# Bioactive Components and Radical Scavenging Activity in Selected Advance Lines of Salt-Tolerant Vegetable Amaranth

**DOI:** 10.3389/fnut.2020.587257

**Published:** 2020-11-30

**Authors:** Umakanta Sarker, Md. Nazmul Hossain, Md. Asif Iqbal, Shinya Oba

**Affiliations:** ^1^Department of Genetics and Plant Breeding, Faculty of Agriculture, Bangabandhu Sheikh Mujibur Rahman Agricultural University, Gazipur, Bangladesh; ^2^Laboratory of Field Science, Faculty of Applied Biological Sciences, Gifu University, Gifu, Japan

**Keywords:** nutraceuticals, pigments, polyphenols and flavonoids, vitamin C, antioxidant activity, HPLC, LC-MS-ESI, abiotic stress-tolerant underutilized vegetables

## Abstract

Four selected advance lines of salt-tolerant vegetable amaranth were evaluated for proximate, nutraceuticals, pigments, phytochemicals, and antioxidants components antioxidants activity in completely randomized block design (RCBD) design in three replicates. Salt-tolerant vegetable amaranth contained adequate carbohydrates, protein, moisture, and dietary fiber. The remarkable contents of iron, manganese, copper, zinc, sodium, molybdenum, boron, potassium, calcium, magnesium, phosphorus, sulfur, betacyanins, betalains, betaxanthins, chlorophylls, ascorbic acid, polyphenols, flavonoids, and antioxidant potentiality were found in salt-tolerant vegetable amaranth. The genotypes LS7 and LS9 had abundant proximate, nutraceuticals, pigments, phytochemicals, and antioxidants compared to the genotypes LS3 and LS5. Salt-tolerant vegetable amaranth demonstrated high content of flavonoid compounds including flavonols such as rutin, kaempferol, isoquercetin, myricetin, hyperoside, and quercetin; flavanol, such as catechin; flavone such as apigenin; and flavanone, such as naringenin. For the first time, we identified one flavonol such as myricetin; one flavanol, such as catechin; one flavone such as apigenin; and one flavanone, such as naringenin in salt-tolerant vegetable amaranth. Across six flavonols, rutin and quercetin were identified as the most prominent compounds followed by isoquercetin and myricetin in selected salt-tolerant vegetable amaranths. Across the genotypes, LS7 exhibited the highest flavonols such as rutin, kaempferol, isoquercetin, myricetin, hyperoside, and quercetin as well as the highest flavanols, such as catechin; flavones such as apigenin; and flavanones, such as naringenin. It revealed from the correlation study that antioxidant components of salt-tolerant vegetable amaranth genotypes exhibited good radical quenching capacity of 2,2′-azino-bis(3-ethylbenzothiazoline-6-sulfonic acid) and 2,2-diphenyl-1-picrylhydrazyl equivalent to Trolox. The two genotypes LS7 and LS9 of vegetable amaranth containing excellent sources of proximate, nutraceuticals, pigments, phytochemicals, and antioxidants components could be used as potent antioxidants to attaining nutrients and antioxidant sufficiency in the saline prone area of the globe. We can extract colorful juice from the genotypes LS7 and LS9 as drink purposes for consuming the nutraceuticals and antioxidant deficient community in the saline prone area around the world. However, further detail experimentation is required to confirm the standardization and stabilization of functional components of vegetable amaranth for extraction of juice as drinks.

## Introduction

Foods' acceptability mostly depends on color, flavor, and taste. For this reason, recently coloring food products have been put forward as they considerably accepted the common interest of the people around the globe. These products interested the consumers in the safety, nutritional, and aesthetic aspects of foods. These products also increase the consumption of natural pigments including betacyanins, betaxanthins, betalains, anthocyanin, amaranthine, chlorophylls, and carotenoids. Vegetable amaranth is a unique source of betalains (betaxanthins and betacyanins) that has important free radical-scavenging activity (1). Betalains could be used as a food colorant in low-acid foods and it has higher pH stability than anthocyanins (2). Amaranthine, a major pigment of betacyanins in vegetable amaranth had very strong antioxidant potentials. It could be used as a substitute source for the well-known betanins from red beets in the food colorants and natural antioxidants (1). Vegetable amaranth has wide adaptability to different abiotic stresses like drought (3–6) and salinity (7–9).

Amaranth (belongs to the family *Amaranthaceae*) is C_4_ and a fast-growing plant with versatile uses such as for ornamental plants, vegetables, and grains. It has wider acclimatization and distributed in America, Africa, Australia, Asia, and Europe. Edible stems and leaves of vegetable amaranth are low-cost vegetables and have abundant protein with important amino acids including methionine and lysine, dietary fiber, carotenoids, vitamin C, minerals, such as calcium, magnesium, potassium, phosphorus, iron, zinc, copper, and manganese (10–15). This genus has many traditional medicinal uses, especially as antiviral, antimalarial, antidiabetic, antibacterial, antihelminthic, and snake antidote (16, 17). It has also abundant antioxidant pigments, such as betacyanins, anthocyanin, betaxanthins, betalains, carotenoids, and chlorophylls (18, 19); and antioxidant phytochemicals, such as vitamin C, phenolic acids, and flavonoids (20). These natural compounds have a remarkable contribution to the industry of food as they scavenge reactive oxygen species (ROS) in the human body and remedy several diseases like cardiovascular diseases, cancer, cataracts, atherosclerosis, retinopathy, arthritis, emphysema, and neurodegenerative diseases (21–24).

Morphologically amaranth is red and green in color (25). Red color amaranth has more pigments like amaranthine, betacyanins, anthocyanin, betaxanthins, carotenoids, and betalains than green color amaranth. Vegetable amaranth (*Amaranthus gangeticus*) has great variability and phenotypic diversity in Asia including Bangladesh and India (26) and has multipurpose uses. The selected genotypes are bright red-violet and maroon in color because of the presence of abundant betalains. It is popular and the cheapest leafy vegetables in Bangladesh and Asia. Its nutritional value, taste, and attractive leaf color has attracted people as a very popular vegetable in the Asian continent and elsewhere. In comparison to lettuce, Amaranth contains 18 times more vitamin A, 13 times more vitamin C, 20 times more calcium and 7 times more iron (27). Salt-tolerant vegetable amaranth leaves contain higher zinc and iron content than cassava leaves (28) and beach pea (29). Jimenez-Aguiar and Grusak (30) reported that potassium, calcium, magnesium, phosphorus, sulfur, manganese, iron, zinc, and copper in the leaves of amaranth were more pronounced than black nightshade, spinach, spider flower, black nightshade, and kale. In Bangladesh and India, Vegetable amaranth is grown year-round and even in the gaps of foliage crops between winter and hot summer (10, 11). Vegetable amaranth leaves inhibit the proliferation of colon (Caco-2) and breast (MCF-7) cancer cell lines and liver (HepG2) exhibit anticancer potential (31).

Recently, we have been exploring salt-tolerant vegetable amaranth genotypes containing high pigments, nutraceuticals, antioxidant phytochemicals, and phenolics of interest for making drinks for the sustainable health benefit of the consumers in the salinity-prone and coastal belt area of the globe. For this purpose, previously, we evaluated germplasms based on salt-tolerance, high yields, and antioxidant potential and four selected advance lines of salt-tolerant vegetable amaranths. It is the first attempt to study the pigments, nutraceuticals, antioxidant phytochemicals, phenolic and flavonoids, and antioxidant capacity in salt-tolerant vegetable amaranth. We ultimately study the possibility of the salt-tolerant genotypes for extracting colorful juice as drink purposes containing abundant pigments, nutraceuticals, antioxidant phytochemicals, phenolics, antioxidant capacity, and flavonoids.

## Materials and Methods

### Experimental Materials

This is the first report on phenolic profiles, antioxidant compositions, and antioxidant capacity in salt-tolerant vegetable amaranth. We previously evaluated several genotypes based on salt tolerance, antioxidant, and yield potentiality to select the best four high-yielding and antioxidant-enriched salt-tolerant genotypes for this experiment.

### Design and Layout

We executed the experiment in three replicates following a completely randomized block design (RCBD) at Bangabandhu Sheikh Mujibur Rahman Agricultural University. Each genotype was grown in a 1 m^2^ experimental plot following 20 cm and 5 cm distances between rows and plants, respectively.

### Intercultural Practices

Recommended compost doses, fertilizer, and appropriate cultural practices were maintained (32). For maintaining the exact spacing of plants in a row, proper thinning was executed. Weeds of experimental plots were regularly removed through proper weeding and hoeing. We provided regular irrigation in the experimental plots for maintaining the proper growth of vegetable amaranth. Leaves from 35-day-old plants were sampled for all biochemical analyses.

### Solvent and Reagents

Solvent: Acetone, hexane, and methanol. Reagents: dithiothreitol (DTT), cesium chloride, HClO_4_, HNO_3_, H_2_SO_4_, ascorbic acid, standard compounds of pure Trolox (6-hydroxy-2, 5, 7, 8-tetramethyl-chroman-2-carboxylic acid), Folin-Ciocalteu reagent, gallic acid, DPPH, rutin, ABTS^+^, 2, 2-dipyridyl, aluminum chloride hexahydrate, potassium acetate, sodium carbonate, and potassium persulfate.

### Estimation of Proximate Composition

ASAE standards were followed to estimate moisture content (3). In triplicates, the fresh samples of vegetable amaranth leaves were oven-dried for 72 h at 103°C. Then the samples were transferred to a desiccator and allowed to stand at room temperature for cooling. A Denver digital balance (USA) was used to record the weights of the samples. AOAC method described by Sarker and Oba (3) was followed to estimate the ash, crude fat, fiber, crude protein contents, and gross energy. The weight of leaf samples was recorded before and after heat treatment (550°C for 12 h) to estimate ash content. Crude fat content was determined according to AOAC method 960.39. Crude protein was assessed by the micro-Kjeldahl method described by Sarker and Oba (3). Finally, nitrogen was multiplied by 6.25 to measure crude protein (AOAC method 976.05). ISO method described by Sarker and Oba (3) was followed to determine fiber content. Powdered leaf samples were boiled for 30 min adding 0.255 M sulfuric acid. The insoluble residue was filtered again, washed, and boiled in 0.313 M sodium hydroxide. After filtering and washing the sample, it was dried at 130 ± 2°C for 2 h. At 350 ± 25°C temperature, the loss of weight was measured. Fiber content was expressed as fresh weight (FW). The total moisture, crude protein, ash, and crude fat (%) were subtracted from 100 for calculating carbohydrate (g 100 g^−1^ FW). A bomb calorimeter was used to measure gross energy according to ISO method 9831 method described by Sarker and Oba (3).

### Estimation of Mineral Composition

The fresh leaf samples of salt-tolerant vegetable amaranth were dried in an oven at 70°C for 24 h. Dried samples were ground in a mill. We determined calcium, potassium, magnesium, phosphorus, sulfur, iron, manganese, copper, zinc, sodium, molybdenum, and boron from powdered leaves following the nitric-perchloric acid digestion method (3). For this digestion, in the presence of carborundum beads, 40 ml HClO_4_ (70%), 400 ml HNO_3_ (65%), and 10 ml H_2_SO_4_ (96%) were added to 0.5 g dried leaf sample. After digestion, the ascorbic acid method was followed to measure P through dilution of the solution appropriately in triplicate. We added ascorbic acid and antimony to the yellow-colored complex solution for converting it to a blue-colored phosphomolybdenum complex. The method of Sarker and Oba (3) was followed to read the absorbance by atomic absorption spectrophotometry (AAS) (Hitachi, Tokyo, Japan) at a wavelength of 285.2 nm (magnesium), 76 6.5 nm (potassium), 880 nm (phosphorus), 258.056 nm (sulfur), 248.3 nm (iron), 422.7 nm (calcium), 279.5 nm (manganese), 213.9 nm (zinc), 324.8 nm (copper), 589 nm (sodium), 313.3 nm (molybdenum), and 430 nm (boron).

### Determination of Chlorophylls

Chlorophyll *ab*, chlorophyll *b*, and chlorophyll *a* were calculated by extracting the fresh leaves in acetone (80%) (3). A spectrophotometer (Hitachi, U-1800, Tokyo, Japan) was used to measure the absorbance at 646 nm for chlorophyll *b* and 663 nm for chlorophyll *a*, respectively. Chlorophylls were calculated as micrograms per gram of FW.

### Betacyanins and Betaxanthins Content Measurement

The fresh leaves were extracted in 80% methyl alcohol having 50 mM ascorbate to measure betacyanins and betaxanthins according to the method of Sarker and Oba (33, 34). A spectrophotometer (Hitachi, U-1800, Tokyo, Japan) was used to measure the absorbance at 540 nm for betacyanins and 475 nm for betaxanthins, respectively. The data were calculated as the ng betanin equivalent per g of FW for betacyanins and ng indicaxanthin equivalent per gram of FW for betaxanthins.

### Estimation of Ascorbic Acid

A Hitachi spectrophotometer (U-1800, Tokyo, Japan) was utilized to estimate ascorbic acid (AsA) and dehydroascorbic acid (DHA) from the fresh leaves. Dithiothreitol (DTT) was used for the sample pre-incubation and reduction of dehydroascorbic acid into ascorbic acid. Ascorbic acid reduced ferric ion to ferrous ion. Reduced ferrous ion forms complexes with 2, 2-dipyridyl (35, 36). We read the absorbance of Fe^2+^ complexes with 2, 2-dipyridyl at 525 nm for estimation of vitamin C through the spectrophotometer (Hitachi, U-1800, Tokyo, Japan). We calculated vitamin C in mg 100 g^−1^ FW.

### Estimation of Total Polyphenols

Extraction of total polyphenols was carried out according to Jimenez-Aguilar and Grusak (30) using 25 mg of fresh sample in 2.5 mL of 1.2 M HCl containing methanol (90%) at 90°C for 2 h in a water bath. With readjusting the volume (2.5 mL), the leaf extract was centrifuged at 7,500 rpm for 20 min. The leaf extracts (100 μL) were added to the Folin-Ciocalteau reagent (2 N, 50 μL); after 5 min, 2 N Na_2_CO_3_ (400 μL) and water (1 mL). The leaf extracts were incubated for 90 min at 37°C. Finally, it was removed to a microplate (flat bottom). In a microplate reader, the absorbance was detected at 740 nm using gallic acid (GAE) as standard μg g^−1^ of FW.

### Estimation of Total Flavonoids

Total flavonoids were extracted and quantfied according to the method described by Jimenez-Aguilar and Grusak (30). Dry leaf samples (100 mg) were mixed with 5 mL methanol (50%) in water and placed for 1 h with ultrasound. The leaf extracts were centrifuged for 10 min at 13,000 g (4°C). The supernatants were then recovered. Flavonoid extracts (400 μL) were homogenized with water (500 μL), 5% NaNO_2_ (60 μL), 10% AlCl3 (140 μL). After 10 min, 1 mM NaOH (400 μL) was added. The leaf extracts were incubated for 10 min at a normal temperature. Finally, it was removed to a flat bottom microplate. The absorbance was read at 500 nm in a microplate reader. Results are expressed in μg of rutin equivalents (RE) per gram of sample DW.

### Radical Quenching Capacity Assay

Fresh leaves were harvested from 35-day-old plants. For the antioxidant capacity assay, the leaves were dried in the air in a shade; 40 ml aqueous methanol (90%) was utilized to extract ground dried leaves (1 g) from each cultivar in a capped bottle (100 ml). A Thomastant T-N22S (Thomas Kagaku Co. Ltd., Japan) shaking water bath was utilized to extract leaf samples for 1 h. An exactly 0.45 μm filter (MILLEX-HV syringe filter, Millipore Corporation, Bedford, MA, USA) was used to filter the homogenized mixture. After centrifugation for 15 min at 10,000 × *g*, the antioxidant capacity was estimated from the filtered extract.

Diphenyl-picrylhydrazyl (DPPH) radical degradation method (37, 38) was used to estimate the antioxidant activity. We added 1 ml DPPH solution (250 μM) to 10 μl extract (in triplicate) in a test tube. After adding 4 ml distilled water the extract was placed in the dark for 30 min. A Hitachi U1800 spectrophotometer (Hitachi, Tokyo, Japan) was used to measure the absorbance at 517 nm. The method of Khanam et al. (39) was followed for ABTS^+^ assay. To prepare two stock solutions separately an ABTS^+^ solution of 7.4 mM and potassium persulfate of 2.6 mM was used. We mixed both solutions in equal proportion to prepare the working solution at room temperature. The working solution was allowed to react in the dark for 12 h; a 150-μl extract was added to 2.85 ml of ABTS^+^ solution and allowed to react in the dark for 2 h. For the preparation of the solution, 1 ml of ABTS^+^ solution was mixed with 60 ml of methanol. A Hitachi spectrophotometer (U1800, Tokyo, Japan) was utilized to take the absorbance against methanol at 734 nm. The inhibition (%) of DPPH and ABTS^+^ corresponding with control was used to determine antioxidant capacity using the equation as follows:

$$\begin{array}{l} \text{Antioxidant activity}\left(\%\right)=(\text{Abs}.\text{ blank }-\text{Abs}.\text{ sample}/\text{Abs}.\\ \;\text{blank})\times 100 \end{array}$$

Where, Abs. blank is the absorbance of the control reaction [10 μl methanol for TAC (DPPH), 150 μl methanol for TAC (ABTS^+^) instead of leaf extract] and Abs. sample is the absorbance of the test compound. Trolox was used as the reference standard, and the results were expressed as μg Trolox equivalent g^−1^ DW.

### Samples Extraction for HPLC and LC-MS Analysis

The fresh leaf samples were extracted by adding 10 ml methanol (80%) containing acetic acid (1%) in 1 g leaves. The mixture was thoroughly homogenized. Then the mixture was kept in a test tube (50 ml) and capped tightly. The test tube was shaken in a shaker (Scientific Industries Inc., USA) for 15 h at 400 rpm. An exactly 0.45 μm filter (MILLEX®-HV syringe filter, Millipore Corporation, Bedford, MA, USA) was used to filter the homogenized mixture. We centrifuged the mixture at 10,000 × *g* for 15 min. The flavonols, flavanols, flavones, and flavanones were analyzed from the final filtrate. We performed all extractions in triplicate independent samples.

### Flavonols, Flavanols, Flavones, and Flavanones Analysis Through HPLC

The method previously described by Sarker and oba (4, 33) was followed to determine flavonols, flavanols, flavones, and flavanones in the fresh leaf sample using HPLC. We equipped the Shimadzu SCL10Avp (Kyoto, Japan) HPLC with a binary pump (LC-10Avp), DGU-14A degasser, and a Shimadzu SPD-10Avp UV–vis detector. A CTO-10AC (STR ODS-II, 150 × 4.6 mm I.D. (Shinwa Chemical Industries, Ltd., Kyoto, Japan) column was used for the separation of flavonols, flavanols, flavones, and flavanones. The binary mobile phase was pumped with solvent A [6% (v/v) acetic acid] in water and solvent B (acetonitrile) at the flow rate of 1 ml/min for 70 min. HPLC system was run using a gradient program with 0–15% acetonitrile for 45 min, 15–30% for 15 min, 30–50% for 5 min, and 50–100% for 5 min; 35°C temperature in the column was maintained with a 10 μl volume of injection (29). We set the detector at 360, 370, and 280 nm, respectively, for continuous monitoring of flavonols, flavanols, flavones, and flavanones. For identification of the compound, we compared the retention time and UV–vis spectra with their respective standards. We confirmed the flavonols, flavanols, flavones, and flavanones through the mass spectrometry assay method. We estimated phenolic compounds as mg kg^−1^ FW. A mass spectrometer (AccuTOF JMS-T100LP, JEOL Ltd., Tokyo, Japan) was fitted with an Agilent 1100 Series HPLC system and a UV–vis detector coupled on-line with an ElectroSpray Ionization (ESI) source to analyze the mass spectrometry with negative ion mode with the column elutes in the range of m/z 0–1,000 and needle voltage at −2000 V. Extract constituents were identified by LC-MS-ESI analysis.

### Quantification of Phenolic Compounds

We used the respective standards of calibration curves to quantify each flavonols, flavanols, flavones, and flavanones. We dissolved 9 flavonol, flavanol, flavone, and flavanone compounds in 80% methanol as stock solutions to the final concentration of 100 mg/ml. Respective standard curves (10, 20, 40, 60, 80, and 100 mg/ml) were used to quantify the individual flavonols, flavanols, flavones, and flavanones compounds with external standards. UV spectral characteristics, retention times, and co-chromatography of samples spiked with commercially available standards were utilized for identification and match the flavonols, flavanols, flavones, and flavanones.

### Statistical Analysis

The Statistix 8 software was used to analyze the data for analysis of variance (ANOVA) (40, 41). Duncan's Multiple Range Test (DMRT) at 1% level of probability was used to compare the means. The results were reported as the mean ± SD of three separate replicates.

## Results

The analysis of variance revealed a wide range of variability of the studied traits regarding selected drought-tolerant leafy vegetable amaranths.

### Composition of Proximate

**Figure 1:**
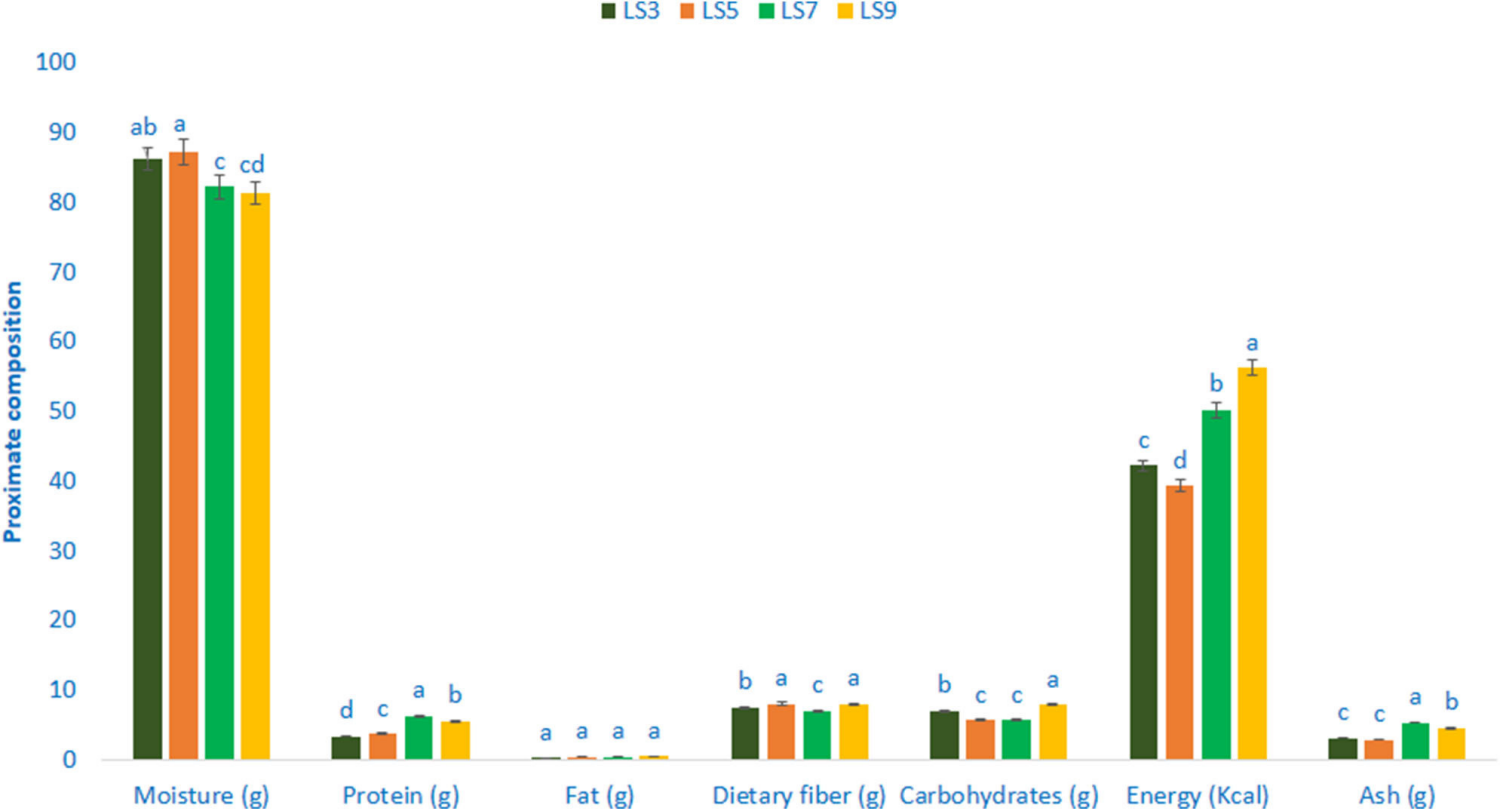
Proximate compositions (g 100 g^−1^ FW) in four selected salt-tolerant vegetable amaranths; different letters are differed significantly by Duncan multiple range test (*P* < 0.01), (*n* = 3).

The composition of the proximate of salt-tolerant vegetable amaranth is shown in Figure 1. The moisture content ranged from 81.35 to 87.24 g 100 g^−1^ FW. The highest moisture content was recorded in LS5 (87.24 g 100 g^−1^ FW), while the lowest moisture content was found in LS9 (81.35 g 100 g^−1^ FW). Salt-tolerant vegetable amaranth leaves exerted significant and very much noticeable variations in protein content. The genotype LS7 showed the highest protein content (6.34 g 100 g^−1^) followed by LS9, whereas, the genotype LS3 had the lowest protein content (3.35 g 100 g^−1^). There were no significant variations in fat content in terms of four selected salt-tolerant vegetable amaranths. The range of fat content was 0.33–0.57 g 100 g^−1^ FW. The genotype LS9 had the highest carbohydrates content (7.93 g 100 g^−1^ FW) followed by LS3, while the carbohydrates content was the lowest in LS5 and LS7 (5.73 and 5.64 g 100 g^−1^ FW, respectively). The genotype LS9 had the highest energy (56.28 kcal 100 g^−1^ FW) followed by LS7, while the lowest energy was obtained from the genotype LS5 (39.28 kcal 100 g^−1^ FW). Ash content was the highest in LS7 (5.36 g 100 g^−1^ FW) followed by LS9, while the lowest ash content was noted in LS5 and LS3 (2.88 and 3.13 g 100 g^−1^ FW). Content of digestible fiber exhibited the least variations in four selected salt-tolerant vegetable amaranths studied. The accession LS5 and LS9 showed the highest dietary fiber content (8.06 and 7.95 g 100 g^−1^ FW) followed by LS3, whereas dietary fiber content was the lowest in LS7 (6.98 g 100 g^−1^ FW).

### Mineral Composition (Macroelements)

**Figure 2:**
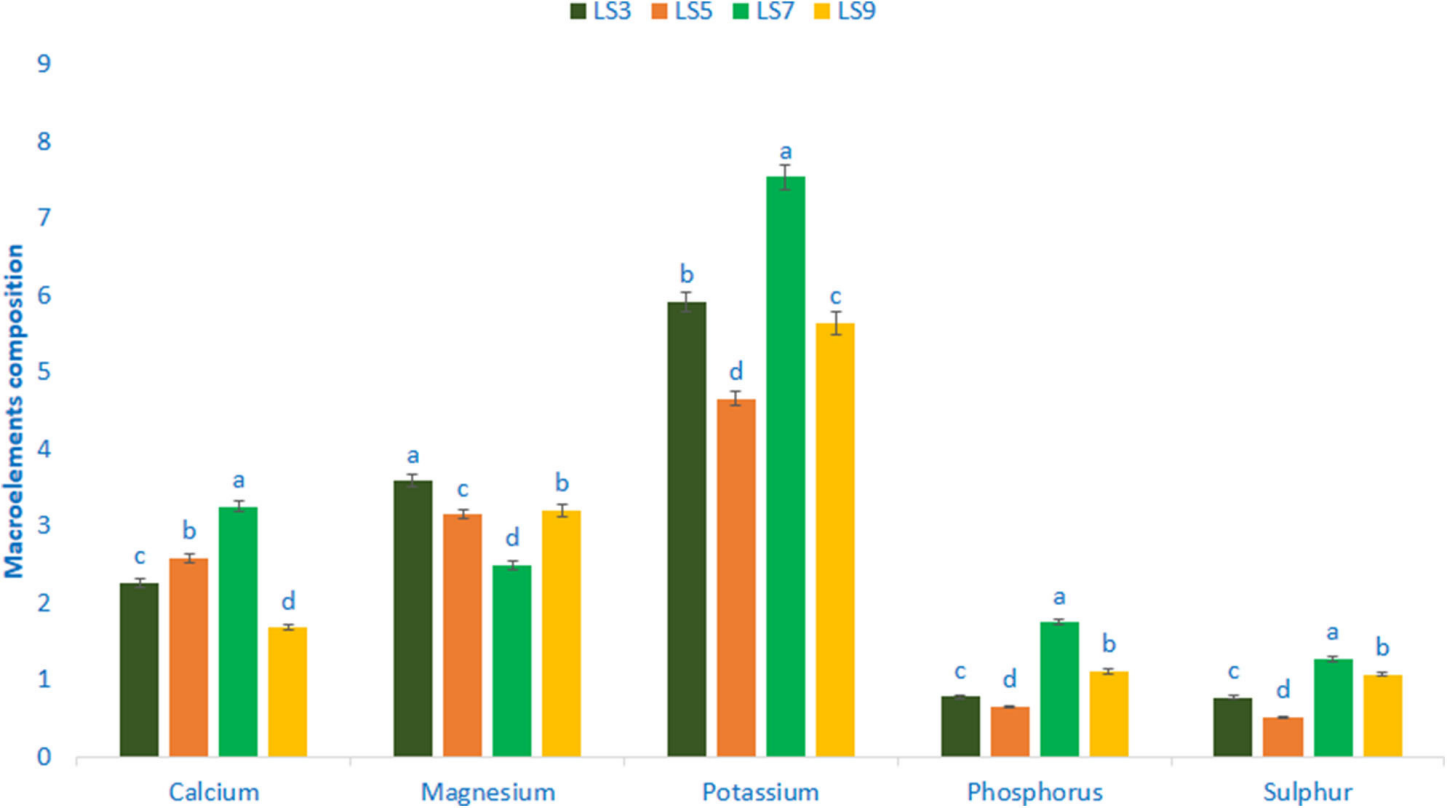
Mineral compositions (Macroelements mg g^−1^ FW,) in four selected salt-tolerant vegetable amaranths; different letters are differed significantly by Duncan multiple range test (*P* < 0.01), (*n* = 3).

Mineral composition (macroelements) of salt-tolerant vegetable amaranth is shown in Figure 2. In this study, the range of potassium content was 4.66 mg g^−1^-7.54 mg g^−1^ FW. The genotypes LS7 had the highest potassium content, while genotype LS5 had the lowest potassium content. Calcium content ranged from 1.68 to 3.25 mg g^−1^ FW. The genotypes LS7 showed the highest calcium content, while the genotype LS9 had the lowest calcium content. Magnesium content was the highest in LS3 (3.59 mg g^−1^ FW) followed by LS5 and LS9. In contrast, the lowest magnesium was recorded in LS7 (2.49 mg g^−1^ FW). Phosphorus and sulfur content of vegetable amaranth leaves ranged from 0.65 to 1.75 and 0.51 to 1.27 mg g^−1^ FW. The genotype LS7 exhibited the highest phosphorus and sulfur content, while the genotype LS5 showed the lowest phosphorus and sulfur content.

### Mineral Composition (Microelements)

**Figure 3:**
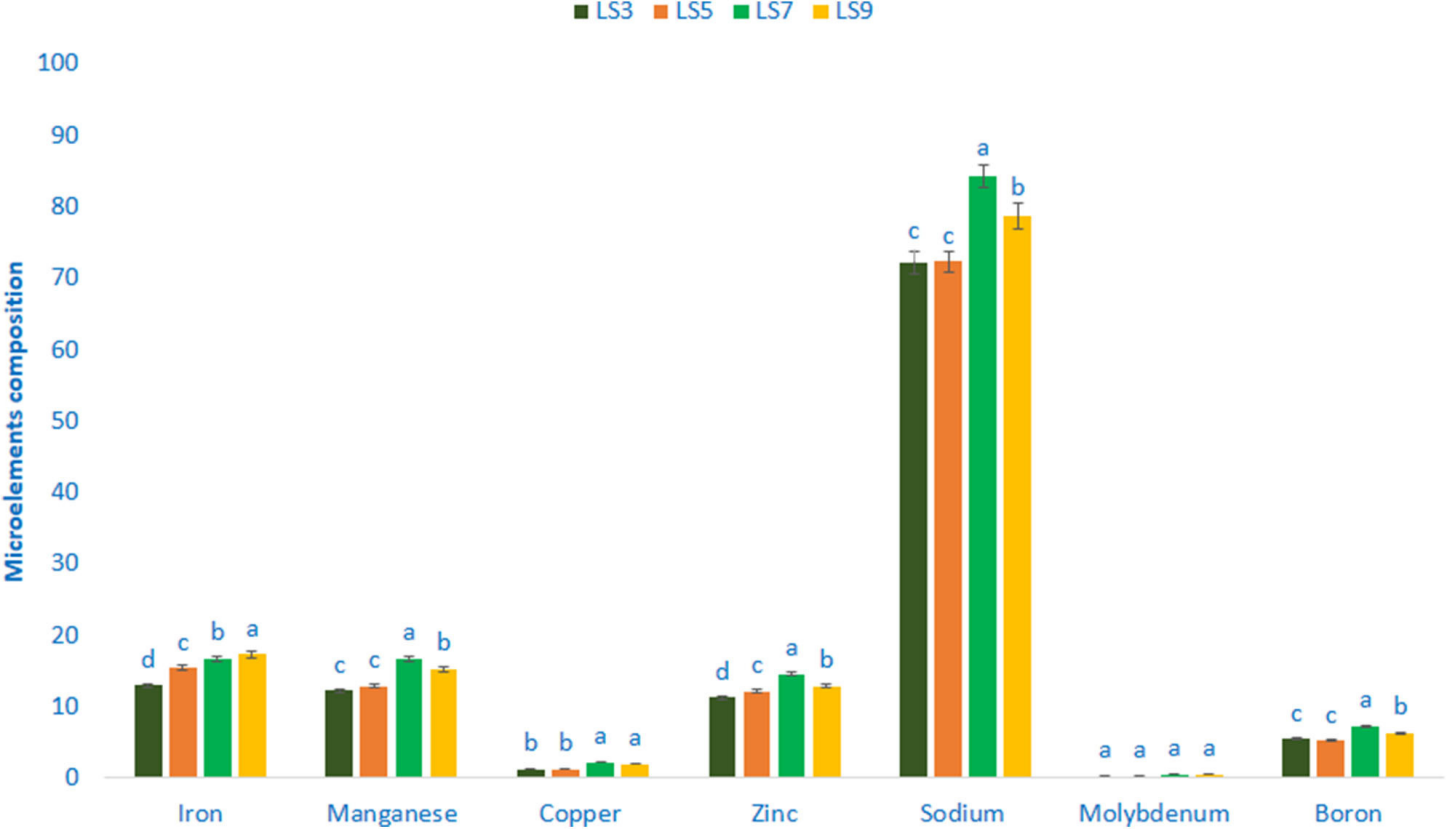
Mineral compositions (Microelements μg g^−1^ FW) in four selected salt-tolerant vegetable amaranths, different letters are differed significantly by Duncan multiple range test (*P* < 0.01), (*n* = 3).

Minerals composition (Microelements) of salt-tolerant vegetable amaranth is shown in Figure 3. Salt-tolerant vegetable amaranth showed remarkable iron and manganese content. The genotype LS9 had the highest iron content (17.35 μg g^−1^ FW) followed by LS7 and LS5, whereas the genotype LS3 showed the lowest iron content (12.99 μg g^−1^ FW). In this study, the range of manganese content was 12.25 μg g^−1^ FW and 16.77 μg g^−1^ FW. The genotype LS7 had the highest manganese content; however, the genotype LS3 had the lowest manganese content. The significant and notable variations of copper content were reported in salt-tolerant vegetable amaranth genotypes (1.27–2.26 μg g^−1^ FW). The copper content was the highest in LS7, followed by LS9; whereas the lowest copper content was obtained from the genotype LS3 and LS5, respectively. Salt-tolerant vegetable amaranth showed remarkable zinc, sodium, and boron content. Zinc, sodium, and boron content ranged from 11.33 to 14.61, 72.24 to 84.29, and 5.27 to 7.36 μg g^−1^ fresh weight, respectively. The genotypes LS7 had the highest zinc, sodium, and boron content, while LS3 showed the lowest zinc and sodium, and LS5 had the lowest boron content. Molybdenum content ranged from 0.26 to 0.57 μg g^−1^ FW. The genotypes LS7 had the highest molybdenum content, while LS3 showed the lowest molybdenum content.

### Pigments Composition

**Figure 4:**
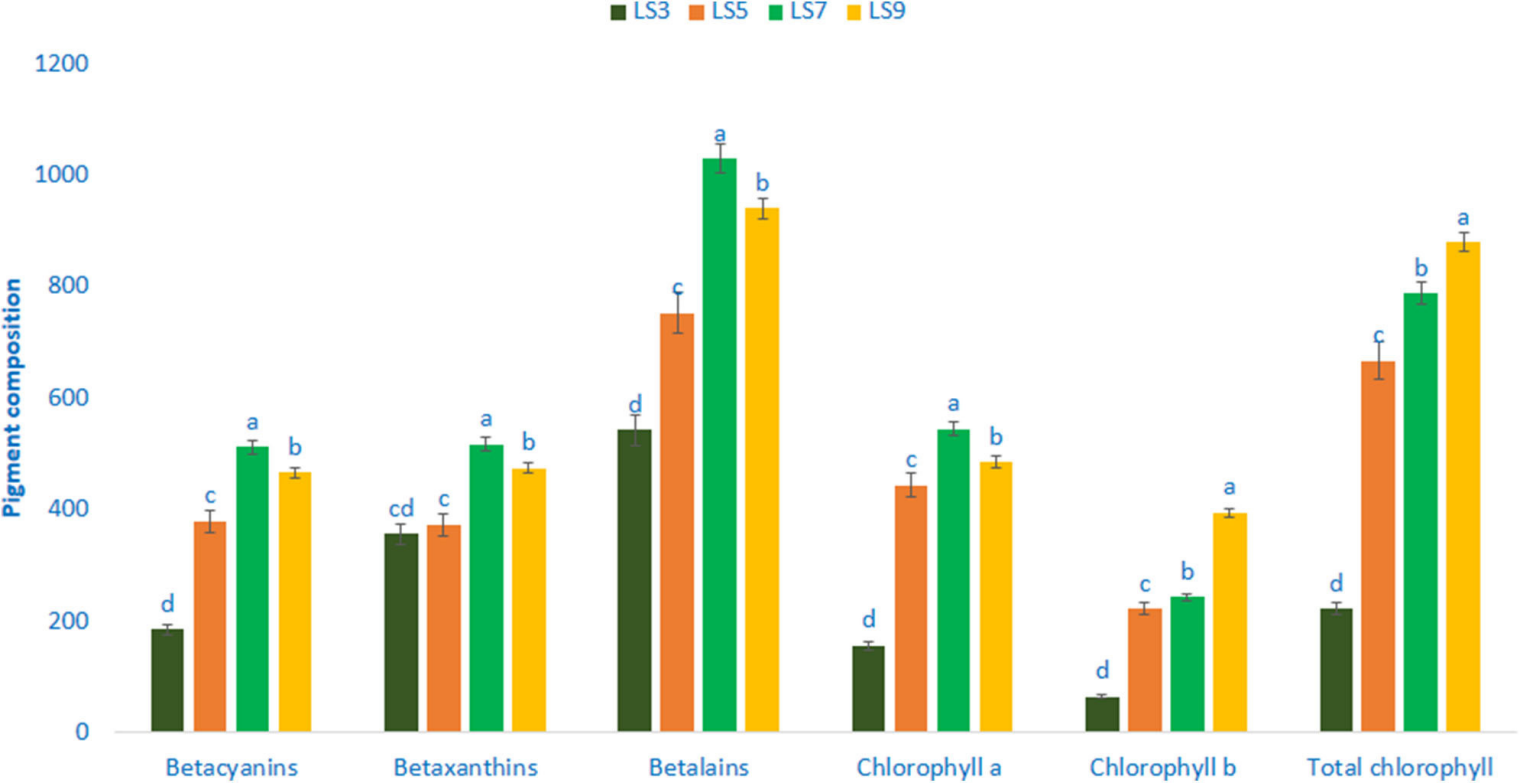
Pigment composition in four selected salt-tolerant vegetable amaranths, betacyanins (ng g^−1^ FW), chlorophyll *a* (μg g^−1^ FW), betaxanthins (ng g^−1^ FW), chlorophyll *b* (μg g^−1^ FW), betalains (ng g^−1^ FW), total chlorophyll (μg g^−1^ FW); different letters in the bar are differed significantly by Duncan multiple range test (*P* < 0.01), (*n* = 3).

Pigments of four selected salt-tolerant vegetable amaranths are shown in Figure 4. Betalains, betaxanthins, and betacyanins varied significantly and remarkably with the genotypes. Betalains, betaxanthins, and betacyanins ranged from 542.35 to 1,029.12, 355.98 to 517.12, and 185.02 to 512.06 ng g^−1^ FW, respectively. The genotype LS7 exhibited the highest betacyanins content, followed by LS9. Conversely, the genotype LS3 had the lowest betacyanins content. Among genotypes, considerable and significant variations were observed in betaxanthins content. Betalains and betaxanthins content were the highest in genotype LS7 followed by LS9. In contrast, genotype LS3 showed the lowest betaxanthins and betalains. The significant and notable variations were noticed for chlorophyll *a* content (156.09–545.06 μg g^−1^ FW). The genotype LS7 had the highest chlorophyll *a* content (545.06 μg g^−1^ FW), whereas the lowest chlorophyll *a* was recorded in LS3 (156.09 μg g^−1^ FW). Similar to chlorophyll *a*, significant and marked differences in chlorophyll *b* content were noted in vegetable amaranth genotypes (64.90–394.35 μg g^−1^ FW). LS9 had the highest chlorophyll *b* content (394.35 μg g^−1^ FW), followed by LS7. Conversely, LS3 had the lowest chlorophyll *b* (64.90 μg g^−1^ FW). Total chlorophyll content showed significant and noticeable variation (221.62–879.42 μg g^−1^ FW). LS7 and LS9 exhibited abundant total chlorophyll content, whereas, the lowest total chlorophyll content was obtained from LS3 (221.62 μg g^−1^ FW).

### Phytochemicals and Antioxidant Capacity

**Figure 5:**
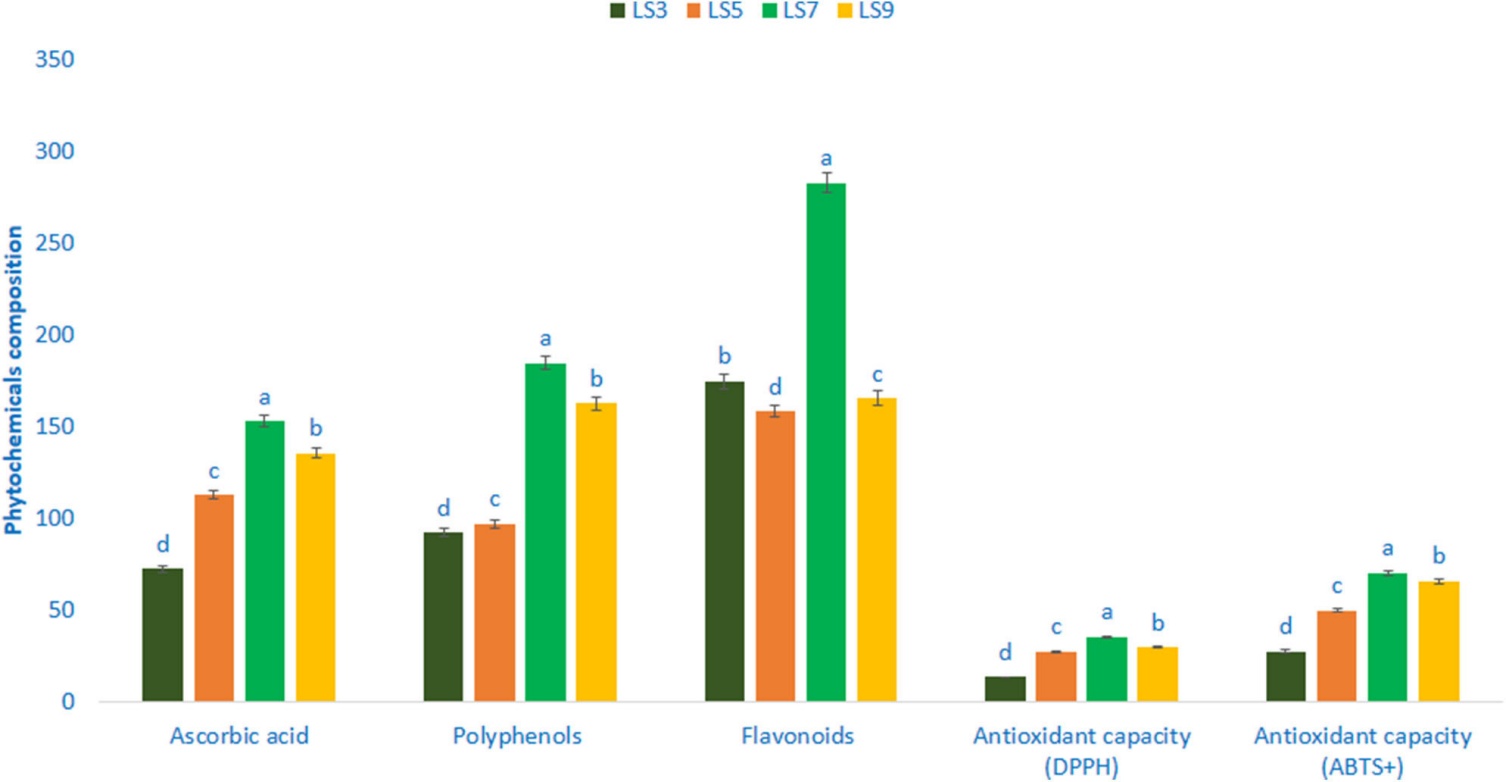
Phytochemical composition and free radical scavenging capacity in four selected salt-tolerant vegetable amaranths, ascorbic acid (mg 100 g^−1^ FW), polyphenols (GAE μg g^−1^ FW), flavonoids (RE μg g^−1^ DW), antioxidant capacity (DPPH) (TEAC μg g^−1^ DW), antioxidant capacity (ABTS^+^) (TEAC μg g^−1^ DW); different letters in the bar are differed significantly by Duncan multiple range test (*P* < 0.01), (*n* = 3).

Polyphenols, flavonoids, ascorbic acid, and antioxidant capacity (AC) varied significantly among the studied salt-tolerant genotypes (Figure 5). Ascorbic acid content ranged from 72.45 mg 100 g^−1^ FW in the genotype LS3 to 152.96 mg 100 g^−1^ FW in the genotype LS7. Polyphenols ranged from 92.26 GAE μg g^−1^ FW (LS3) to 184.76 GAE μg g^−1^ FW (LS7). The genotype LS7 had the highest polyphenols followed by LS9. Flavonoids exhibited much noticeable variation in terms of genotypes, which ranged from 158.34 RE μg g^−1^ DW in the genotype LS5 to 282.87 RE μg g^−1^ DW in the genotype LS7. AC (DPPH) ranged from 13.35 TEAC μg g^−1^ DW (LS3) to 35.36 TEAC μg g^−1^ DW (LS7). The highest AC (DPPH) was recorded in the genotype LS7 followed by LS9 and LS5. In contrast, LS3 had the lowest AC (DPPH). AC (ABTS^+^) ranged from 27.62 TEAC μg g^−1^ DW to 70.24 TEAC μg g^−1^ DW. The salt-tolerant vegetable amaranth genotype LS7 had the highest AC (ABTS^+^) followed by LS9. In contrast, AC (ABTS^+^) was the lowest in LS3.

### Flavonols, Flavanols, Flavones, and Flavanones

**Figure 6:**
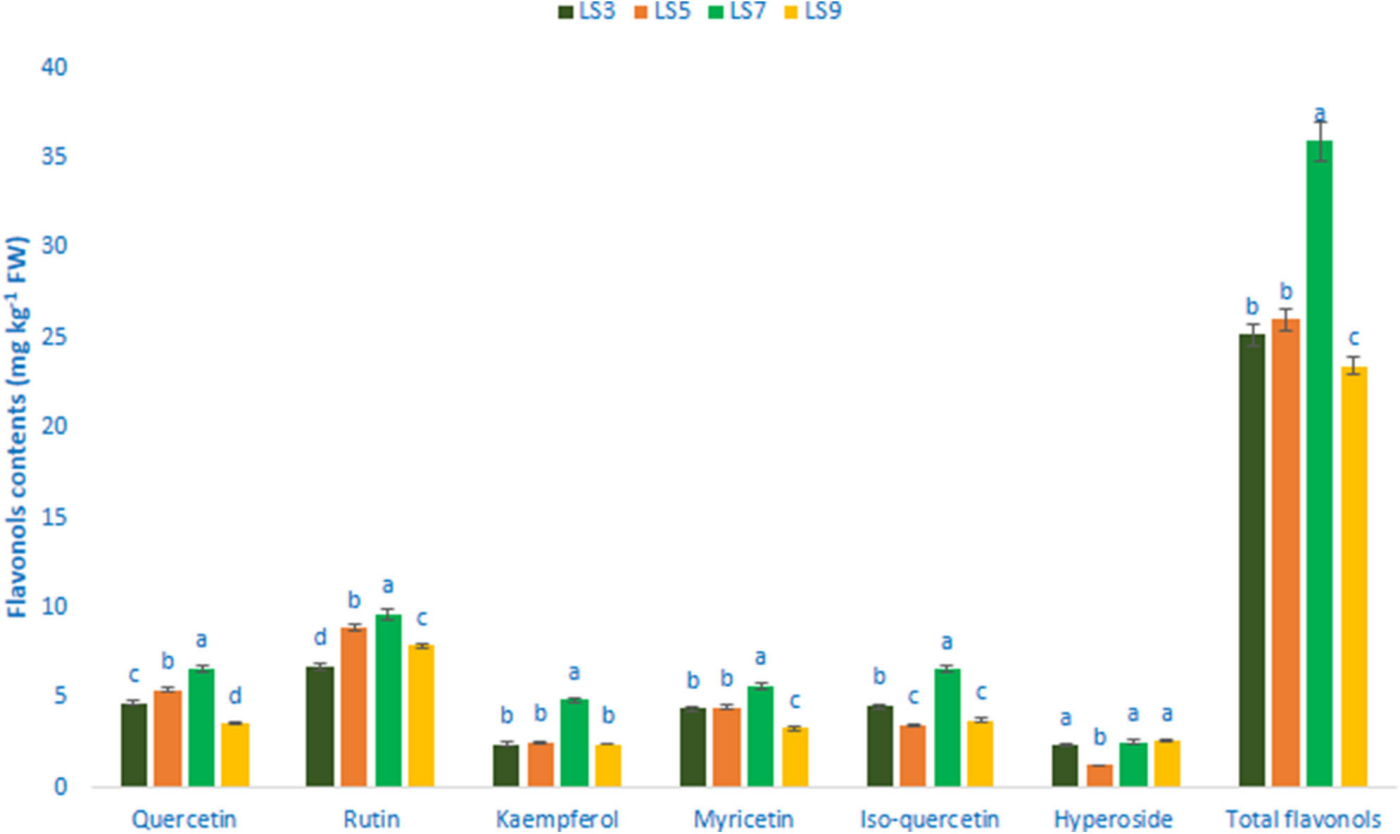
Flavonol contents (mg kg^−1^ FW) in four selected salt-tolerant vegetable amaranths; different letters in the bar are differed significantly by Duncan multiple range test (*P* < 0.01), (*n* = 3).

**Figure 7:**
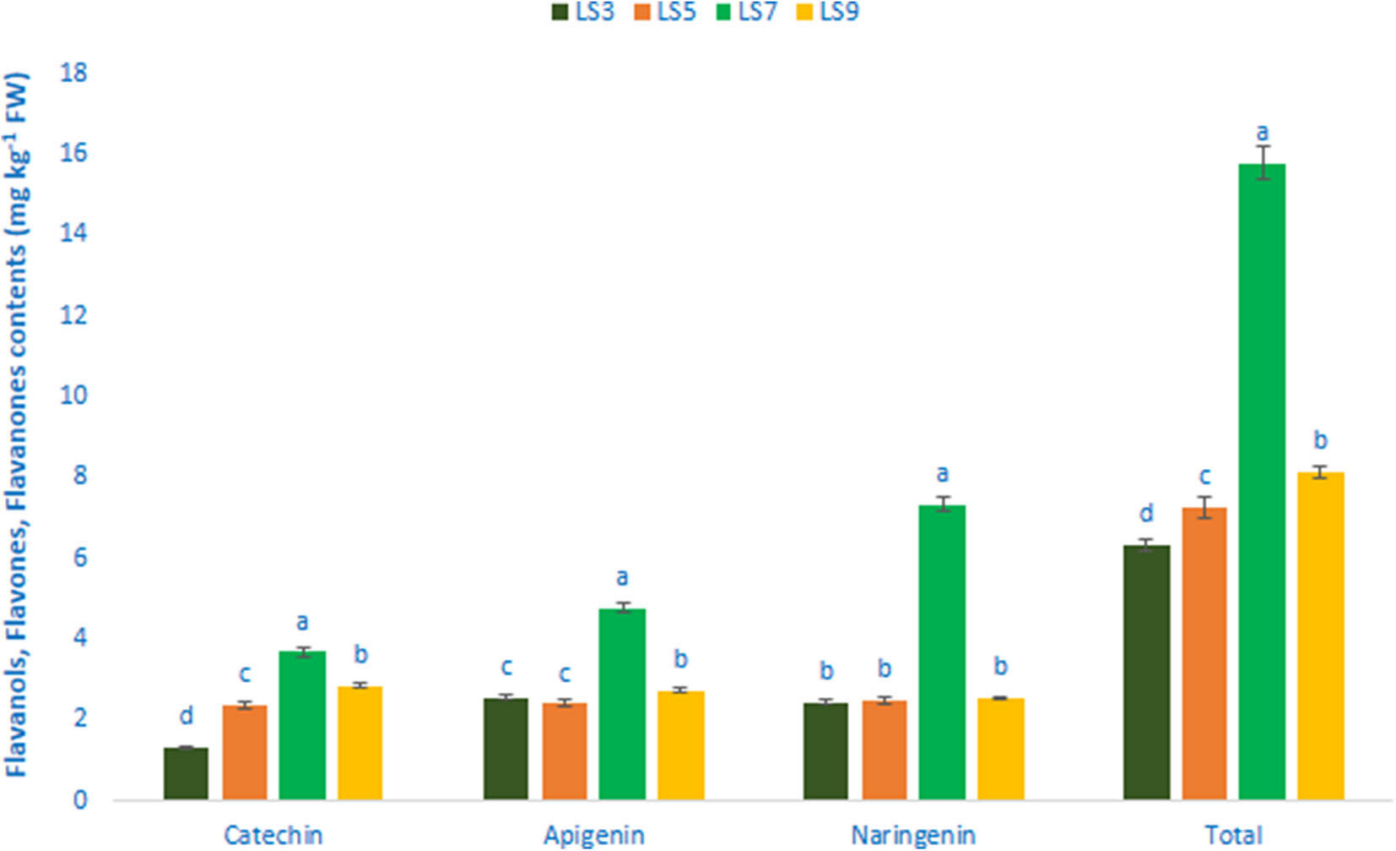
Flavanol, flavone, and flavanone content (mg kg^−1^ FW) in four selected salt-tolerant vegetable amaranths; different letters in the bar are differed significantly by Duncan multiple range test (*P* < 0.01), (*n* = 3).

Table 1 shows the data on main fragment ions in MS^2^, identified compounds, the molecular ion, λ_max_, and retention time. The liquid chromatography separated values of flavonols, flavanols, flavones, and flavanones compounds from four salt-tolerant leafy vegetable amaranths (LS3, LS5, LS7, and LS9) were compared with standard masses of flavonols, flavanols, flavones, and flavanones compounds through the respective peaks of the compounds. Nine flavonoids compounds were determined in salt-tolerant vegetable amaranth including six flavonols, such as rutin, kaempferol, isoquercetin, myricetin, hyperoside, and quercetin, one flavanol, such as catechin, one flavone such as apigenin, and one flavanone, such as naringenin. For the first time, we identified one flavonols such as myricetin, one flavanol, such as catechin, one flavone such as apigenin, and one flavanone, such as naringenin in salt-tolerant vegetable amaranth. Figure 6 showed the identified flavonols compounds and Figure 7 showed the identified flavanols, flavones, and flavanones compounds of leaves of four selected salt-tolerant vegetable amaranths. Across four principal groups of compounds, the most identified pronounced compounds in four selected salt-tolerant vegetable amaranths were observed in the following order: flavavanones > flavones > flavanols (Figures 6, 7). Across six flavonols, rutin and quercetin were identified as the most prominent compounds followed by isoquercetin and myricetin in selected salt-tolerant vegetable amaranths. Across the genotypes, LS7 exhibited the highest flavonols such as rutin, kaempferol, isoquercetin, myricetin, hyperoside, and quercetin. LS5 contained high total flavonols which were statistically similar to LS3, while LS9 demonstrated the lowest flavonols. Rutin, kaempferol, isoquercetin, myricetin, hyperoside, and quercetin of selected salt-tolerant vegetable amaranths varied from 6.75 to 9.62, 2.42 to 4.88, 3.45 to 6.58, 3.28 to 5.68, 1.25 to 2.58, and 3.55 to 6.62 mg kg^−1^ FW, respectively (Figure 6). LS7 exhibited the highest flavanols, such as catechin, flavones such as apigenin, and flavanones, such as naringenin followed by LS9 (Figure 7). In contrast, LS3 showed the minimum flavanols, such as catechin. LS5 showed the minimum flavones such as apigenin which was statistically similar to LS3. Similarly, LS3 showed the minimum flavanones, such as naringenin which as statistically similar to LS5 and LS9 (Figure 7).

**Table 1:** Retention time (Rt), wavelengths of maximum absorption in the visible region (λ_max_), mass spectral data and tentative identification of flavonols, flavanols, flavones, and flavanones in four selected salt-tolerant vegetable amaranths.

**Peak no**	**Rt (min)**	**λ_max_ (nm)**	**Molecular ion [M–H]^**−**^ (m/z)**	**MS^**2**^ (m/z)**	**Identity of tentative compounds**
1	4.58	370	626.1882	626.2714	Myricetin-3-*O*-rutinoside
2	7.55	370	301.0426	301.0421	2-(3,4-dihydroxy phenyl)-3,5,7-trihydroxychromene-4-one
3	15.47	370	270.3432	270.3324	4′,5,7-Trihydroxyflavone, 5,7-Dihydroxy-2-(4-hydroxyphenyl)-4-benzopyrone
4	17.84	370	593.5312	593.3412	kaempferol-3-*O*-rutinoside
5	23.91	280	290.2287	290.2175	(2R-3S)-2-(3,4-dihydroxyphenyl)-3,4-dihydro-2-chromene-3,5,7-triol
6	26.74	280	271.0812	271.1621	Naringenin
7	54.36	360	463.3215	463.3287	Quercetin-3-*O*-glucoside
8	53.35	360	463.4621	463.5325	Quercetin-3-*O*-galactoside
9	53.36	360	609.3698	609.3574	Quercetin-3-*O*-rutinoside

### Correlation Coefficient Analysis

Correlation of antioxidant pigments and phytochemicals of salt-tolerant vegetable amaranth are shown in Table 2. Highly significant positive associations of betalains, betaxanthins, betacyanins, total chlorophyll, chlorophyll *b*, and chlorophyll *a* were exhibited among pigments and with AC (ABTS^+^), AC (DPPH), ascorbic acid, polyphenols, and flavonoids. Ascorbic acid exerted significant associations with all traits along with AC (ABTS^+^) and AC (DPPH). The significant associations of polyphenols and flavonoids were observed with AC (ABTS^+^) and AC (DPPH). Similarly, a significant relationship of AC (ABTS^+^) with AC (DPPH) validated antioxidant activity measurement of different methods in salt-tolerant vegetable amaranth.

**Table 2:** The correlation coefficient for pigments, phytochemicals, and antioxidant capacity in four selected salt-tolerant vegetable amaranths.

	**Betaxanthins (ng g^**−1**^)**	**Betalains (ng g^**−1**^)**	**Chl *a* (μg g^**−1**^)**	**Chl *b* (μg g^**−1**^)**	**T chl (μg g^**−1**^)**	**Ascorbic acid (mg 100 g^**−1**^)**	**Polyphenols (GAE μg g^**−1**^ FW)**	**Flavonoids (RE μg g^**−1**^ DW)**	**AC (DPPH)**	**AC (ABTS^**+**^)**
Betacyanins	0.96^**^	0.94^**^	0.97^**^	0.95^**^	0.98^**^	0.78^**^	0.88^**^	0.96^*^	0.88^*^	0.89^*^
Betaxanthins		0.96^**^	0.92^**^	0.94^**^	0.97^**^	0.76^**^	0.81^**^	0.95^*^	0.91^*^	0.93^*^
Betalains			0.94^**^	0.86^**^	0.94^**^	0.78^**^	0.89^**^	0.93^*^	0.96^*^	0.98^**^
Chl *a*				0.96^**^	0.97^**^	0.75^**^	0.88^**^	0.96^*^	0.82^**^	0.95^**^
Chl *b*					0.97^**^	0.84^**^	0.84^**^	0.98^**^	0.93^*^	0.92^**^
T Chl						0.82^**^	0.87^**^	0.87^**^	0.89^*^	0.94^*^
Ascorbic acid							0.88^**^	0.95^**^	0.97^**^	0.88^**^
Polyphenols								0.91^**^	0.88^**^	0.87^**^
Flavonoids									0.88^*^	0.96^*^
AC (DPPH)										0.97^**^

Chl a, Chlorophyll a; Chl b, Chlorophyll b; T chl, Total chlorophyll; Polyphenols (GAE μg g^−1^ FW); Flavonoids (RE μg g^−1^ DW); AC (DPPH), Antioxidant capacity (DPPH) (TEAC μg g^−1^ DW); AC (ABTS^+^), Antioxidant capacity (ABTS^+^) (TEAC μg g^−1^ DW);

* *significant at 5% level*,

** *significant at 1% level, (n = 3)*.

## Discussion

The analysis of variance revealed a wide range of variability of the studied traits regarding selected drought-tolerant leafy vegetable amaranths. A wide range of variability was also reported in red and green color amaranth (33), rice (42–56), maize (57–59), and coconut (60, 61). The lowest moisture content was noted in the salt-tolerant vegetable amaranth genotypes LS9 and LS7. As leaf higher dry matter obtained from lower moisture contents, two genotypes (19–18% dry matter) had considerable dry biomass. The maturity is directly interrelated to the moisture content of leaves. The results obtained in this study were corroborated to the reports of *A. tricolor* and sweet potato leaves by Sarker and Oba (3) and Sun et al. (62), respectively. Salt-tolerant vegetable amaranth leaves exerted significant and very much noticeable variations in protein content. Poor people and vegetarians of the developing countries mainly depend on vegetable amaranth for their protein source. The protein content of salt-tolerant vegetable amaranth was much higher as compared to *A. tricolor* (1.26%) in our earlier study (11). There were no significant variations in fat content in terms of four selected salt-tolerant vegetable amaranths. Our results were corroborated with the results of Sarker and Oba (3) and Sun et al. (62) in *A. tricolor* and leaves of sweet potato, respectively. They reported that fat influences the cell function, covering the organs of the body, and upholding the temperature of the body. Fats have abundant omega-6 and omega-3 fatty acids. Fats play a significant role in digestion, absorption, and transport of vitamins E, D, A, and K that are soluble in fats.

The salt-tolerant vegetable amaranth genotypes showed considerable dietary fiber. Dietary fiber remarkably contributed to the cure of constipation, increment of digestibility, and palatability (13). It revealed from our results that leaves of salt-tolerant vegetable amaranth have abundant protein, moisture, carbohydrates, and digestible fiber. The results of this study corroborated with the results of our earlier study (3). The digestible fiber and carbohydrates contents obtained from the advance lines were corroborated with our previous studies of red morph amaranth (63), weedy amaranth (64), green morph amaranth (65), stem amaranth (66), and *A. blitum* (67). However, dry matter contents of these advance lines were greater than the dry matter contents of red morph amaranth (63), weedy amaranth (64), green morph amaranth (65), stem amaranth (66), and *A. blitum* (67). Except for weedy amaranth, protein contents of these advance lines were greater than the protein contents of red morph amaranth (63), green morph amaranth (65), stem amaranth (66), and *A. blitum* (67).

In our present study, we found remarkable potassium (7.54 mg g^−1^), calcium (3.25 mg g^−1^) magnesium (3.59 mg g^−1^) phosphorus (1.75 mg g^−1^), and sulfur (1.27 mg g^−1^) in salt-tolerant vegetable amaranth. Chakrabarty et al. (15) in *A. lividus* and Sarker and Oba (3) in *A. tricolor* also observed similar results. Jimenez-Aguiar and Grusak (30) noted abundant potassium, calcium, magnesium, phosphorus, and sulfur in different amaranths. They also noticed that amaranth potassium, calcium, magnesium, phosphorus, and sulfur were much pronounced than black nightshade, spinach, spider flower, and kale. Salt-tolerant vegetable amaranth leaves contained higher zinc and iron content than the cassava leaves (28) and beach pea (29). In this study, we found remarkable iron (17.35 μg g^−1^), manganese (16.77 μg g^−1^), copper (2.26 μg g^−1^), zinc (14.61 μg g^−1^), sodium (84.29 μg g^−1^), molybdenum (0.57 μg g^−1^), and boron (7.36 μg g^−1^) in salt-tolerant vegetable amaranth. Jimenez-Aguiar and Grusak (30) noted abundant iron, manganese, copper, zinc sodium, molybdenum, and boron in different amaranths. They also noticed that manganese, iron, zinc, and copper in the leaves of amaranth were pronounced than black nightshade, spinach, spider flower, black nightshade, and kale. Potassium contents obtained from these advance lines were corroborated with our previous studies of green morph amaranth (65), while calcium contents recorded in these advance lines were greater than red morph amaranth (63), stem amaranth (66), and *A. blitum* (67). We observed high phosphorus and sodium in the current investigation than our weedy amaranth (64). Similarly, iron, zinc, and magnesium obtained from the current investigation were much pronounced than our earlier investigation in red morph amaranth (63), green morph amaranth (65), stem amaranth (66), and *A. blitum* (67). We obtained high copper than our earlier study of green morph amaranth (65) and high manganese than weedy amaranth (64), green morph amaranth (65). Hence, these selected salt-tolerant advance lines could contribute as high minerals enriched genotypes than our previously tested amaranth genotypes.

In this study, we found remarkable total chlorophyll (879.45 μg g^−1^ FW), chlorophyll *a* (545.06 μg g^−1^ FW), and chlorophyll *b* (394.35 μg g^−1^ FW) in salt-tolerant vegetable amaranth, whereas, Khanam and Oba (68) observed comparatively lower chlorophyll content in *A. tricolor*. On the other hand, Khanam and Oba (68) observed a more or less similar trend in betacyanin, betalain, chlorophyll, and betaxanthin content of red and green amaranth. The genotype LS7 and LS9 had abundant betacyanin, betalain, chlorophyll, and betaxanthin content indicating the presence of the high antioxidant activity. The genotype LS7 and LS9 had abundant betacyanins, betalains, chlorophylls, and betaxanthins among leafy vegetables that have important free radical-scavenging activity (1). Presence of high betacyanins, betalains, chlorophylls, and betaxanthins in vegetable amaranth genotype LS7 and LS9 is an important parameter for consumers, having an essential role in detoxification of ROS in the human body and preventing many degenerative human diseases and antiaging (22, 24). Total chlorophyll, chlorophyll *a*, chlorophyll *b*, betacyanins, betalains, and betaxanthins content obtained from these advance lines were greater than red morph amaranth (63), green morph amaranth (65), stem amaranth (66), weedy amaranth (64), and *A. blitum* (67). Hence, these selected salt-tolerant advance lines could contribute as high antioxidant pigments enriched genotypes than our previously tested amaranth genotypes.

Salt-tolerant vegetable amaranth genotype LS7 and LS9 had high polyphenols, flavonoids, ascorbic acid, and antioxidant capacity (AC). Our results were corroborated with the results of Khanam and Oba (68) where they observed higher polyphenols, flavonoids, and AC content in the red amaranth genotype compared to green amaranth. Salt-tolerant vegetable amaranth LS7 and LS9 contained higher ascorbic acid, polyphenols, flavonoids, and AC compared to the genotype LS3 and LS5. Hence, these antioxidant phytochemicals of salt-tolerant vegetable amaranth genotypes could be an important parameter for consumers, playing a crucial role in detoxification of ROS in the human body and preventing antiaging and many degenerative human diseases (22, 24). Our result showed that salt-tolerant vegetable amaranth genotypes contained antioxidant phytochemicals such as ascorbic acid, polyphenols, flavonoids, and AC among leafy vegetables that have the important scavenging activity of free radicals (1). The ascorbic acid and total polyphenols obtained from these advance lines were greater than our previous studies of green morph amaranth (65) and weedy amaranth (64), while total flavonoids recorded in these advance lines were greater than our previous studies of green morph amaranth (65) and red morph amaranth (63). Antioxidant capacity in DPPH obtained from these advance lines was greater than our previous studies of red morph amaranth (63) and antioxidant capacity in ABTS^+^ obtained from these advance lines was greater than our previous studies of red morph amaranth (63), green morph amaranth (65), and stem amaranth (66). Hence, these selected salt-tolerant advance lines could contribute as high vitamin C, polyphenols, flavonoids, and antioxidants enriched genotypes than our previously tested amaranth genotypes.

We observed considerable pigments including betacyanins, betalains, betaxanthins, chlorophylls, and antioxidant phytochemicals such as ascorbic acid, polyphenols, antioxidant potentiality, and flavonoids in salt-tolerant vegetable amaranth genotypes. Our results were fully in agreement to the results of Khanam and Oba (68) where they observed higher AC, betacyanins, flavonoids, betalains, betaxanthins, and polyphenols content in the red amaranth genotype compared to green amaranth. Pigments such as betalains (1,029.12 ng g^−1^), betacyanins (512.06 ng g^−1^), betaxanthins (517.12 ng g^−1^), total chlorophyll (879.45 μg g^−1^ FW), chlorophyll *a* (545.06 μg g^−1^ FW), chlorophyll *b* (394.35 μg g^−1^ FW), and antioxidant phytochemicals such as AC (ABTS^+^) (70.24 TEAC μg g^−1^ DW), flavonoids (282.87 RE μg g^−1^ DW), and AC (DPPH) (35.36 TEAC μg g^−1^ DW) obtained in this study, more or less similar to the findings in *A. tricolor* of Khanam et al. (39), whereas polyphenols obtained in our study were much prominent than the findings in *A. tricolor* of Khanam et al. (39). The genotypes LS7 and LS9 had high pigments such as betacyanins, betalains, betaxanthins, chlorophylls, and antioxidant phytochemicals such as ascorbic acid, polyphenols, flavonoids, and AC.

In this study, we found plentiful protein, carbohydrates, nutraceuticals, and digestible fiber, moisture, remarkable pigments profile such as betacyanins, betalains, betaxanthins, chlorophylls, antioxidant phytochemicals such as ascorbic acid, polyphenols, flavonoids, and antioxidant potential in salt-tolerant vegetable amaranth genotypes. We obtained corroborative results compared to the results of Khanam et al. (39) where they observed higher AC, betacyanin, flavonoid, betalain, betaxanthin, and polyphenols content in the red amaranth. The genotypes LS7 and LS9 had abundant carbohydrates, protein, moisture, and dietary fiber, nutraceuticals, pigments, antioxidant phytochemicals, flavonoids, and antioxidant potentials. The genotypes LS7 and LS9 could be used as antioxidant profile enriched high-yielding varieties as drink purposes. It revealed from the investigation that these two genotypes contained adequate polyphenols, flavonoids, ascorbic acid, pigments, and antioxidant potentials that have prospects for extracting colorful juice for drinking purposes as well as for consuming the nutraceuticals and antioxidant-deficient community in the saline prone area of the world.

Salinity stress induces the ROS accumulation in plants that alters the biosynthesis of flavonoids (69) to mitigate the damaging effects of ROS and to adjust unfavorable stress conditions (70). These flavonoid compounds act as non-enzymatic antioxidants to alleviate the negative effect of ROS in plants through quenching these free radicals (71). Abiotic stress facilitates flavonoid biosynthesis in higher concentration to adjust oxidative stress in plants because of their high antioxidant potentiality (72). Furthermore, salinity stress highly accelerates the activity of biosynthesis pathway genes such as *TT3, TT4, TT5, TT6, TT7, TT8, TT9, TT18, FLS*, and *F60H1* that are involved in the biosynthesis of flavanone, flavone, flavonol, anthocyanin, and its derivatives (73–75). In addition, the regulatory gene *TT8* stimulates many flavonoid biosynthesis pathways genes and facilitates the accumulation of more antioxidant flavonoid compounds in plants (76). The data of foxtail millet roots showed that 17 flavonoid biosynthesis genes were significantly up-regulated 2–11-fold under salinity. In keeping with gene expressions, the over-accumulation of 27 flavonoids was obtained under salt tolerance (77).

Nine flavonoid compounds were determined in salt-tolerant vegetable amaranth including six flavonols, such as rutin, kaempferol, isoquercetin, myricetin, hyperoside, and quercetin; one flavanol, such as catechin; one flavone such as apigenin; and one flavanone, such as naringenin. For the first time, we identified one flavonol such as myricetin; one flavanol, such as catechin; one flavone such as apigenin; and one flavanone, such as naringenin in salt-tolerant vegetable amaranth. Khanam et al. (39) and Khanam and Oba (68) noticed three flavonols such as isoquercetin, rutin, and hyperoside in red and green amaranth. In the leaf, stalks, flowers, sprouts, and the seed of *A. cruentus, A. caudatus*, and *A. hypochondriacus*, Li et al. (78) observed three flavonols, such as kaempferol, rutin, and quercetin. Three flavonoids including isovitexin, vitexin, and rutin were reported in the seeds and sprouts of *A. cruentus* (79). Across four principal groups of compounds, the most identified pronounced compounds in four selected salt-tolerant vegetable amaranths were observed in the following order: flavonols > flavanones > flavones > flavanols. Across six flavonols, rutin and quercetin were identified as the most prominent compounds followed by isoquercetin and myricetin in selected salt-tolerant vegetable amaranths. Across the genotypes, LS7 exhibited the highest flavonols such as rutin, kaempferol, isoquercetin, myricetin, hyperoside, and quercetin. LS5 contained high total flavonols which were statistically similar to LS3, while LS9 demonstrated the lowest flavonols. Quercetin and hyperoside of our selected salt-tolerant leafy vegetable amaranths were higher than the content of quercetin and hyperoside reported by Khanam et al. (39) in *A. tricolor* genotypes. The varietal differences, and differential geographic locations, climatic and edaphic conditions, and cultural managements may have played a major contribution in securing higher quercetin and hyperoside in our salt-tolerant vegetable amaranth genotypes in comparison with the results of Khanam et al. (39). LS7 exhibited the highest flavanols, such as catechin; flavones such as apigenin; and flavanones, such as naringenin followed by LS9.

Highly significant positive associations of betalains, betaxanthins, betacyanins, total chlorophyll, chlorophyll *b*, and chlorophyll *a* were exhibited among pigments and with AC (ABTS^+^), AC (DPPH), ascorbic acid, polyphenols, and flavonoids. Pigments of salt-tolerant vegetable amaranth (betalains, betaxanthins, chlorophylls, and betacyanins) showed strong antioxidant activity as all the pigments exhibited significant associations with AC (ABTS^+^) and AC (DPPH). Ascorbic acid exerted significant associations with all traits along with AC (ABTS^+^) and AC (DPPH). The significant positive associations of ascorbic acid with AC (ABTS^+^) and AC (DPPH) also suggested a strong antioxidant activity. The significant associations of polyphenols and flavonoids were observed with AC (ABTS^+^) and AC (DPPH) indicating the strong antioxidant capacity of phenolics and flavonoids in salt-tolerant vegetable amaranth. The TPC, TFC, and TAC of salt-induced purslane and amaranth corroborated with the results of the present investigation (80, 81). Similarly, the significant relationship of AC (ABTS^+^) with AC (DPPH) validated the antioxidant activity measurement of different methods in salt-tolerant vegetable amaranth.

## Conclusion

Salt-tolerant vegetable amaranth genotypes contained ample proximate, pigments, nutraceuticals, and phytochemicals such as protein, carbohydrates, moisture, dietary fiber, polyphenols, minerals, betaxanthins, flavonoids, betacyanins, betalains, and chlorophylls. Salt-tolerant vegetable amaranth genotypes LS7 and LS9 had greater proximate, nutraceuticals, pigments, antioxidant phytochemicals, and antioxidant activity compared to the genotype LS3 and LS5. Nine flavonoids compounds were determined in salt-tolerant vegetable amaranth including six flavonols, such as rutin, kaempferol, isoquercetin, myricetin, hyperoside, and quercetin; one flavanol, such as catechin; one flavone such as apigenin; and one flavanone, such as naringenin. For the first time, we identified one flavonol such as myricetin; one flavanol, such as catechin; one flavone such as apigenin; and one flavanone, such as naringenin in salt-tolerant vegetable amaranth. Across six flavonols, rutin and quercetin were identified as the most prominent compounds followed by isoquercetin and myricetin in selected salt-tolerant vegetable amaranths. Across the genotypes, LS7 exhibited the highest flavonols such as rutin, kaempferol, isoquercetin, myricetin, hyperoside, and quercetin as well as the highest flavanols, such as catechin; flavones such as apigenin; and flavanones, such as naringenin. The correlation study revealed that all antioxidant constituents of salt-tolerant vegetable amaranth had strong antioxidant activity. It revealed from the study that salt-tolerant vegetable amaranth genotypes LS7 and LS9 exhibited excellent sources of proximate, nutraceuticals, pigments, antioxidant phytochemicals, and antioxidant activity that offered huge prospects for nutritional and health-boosting effects. We can extract colorful juice from the genotypes LS7 and LS9 as drink purposes for consuming the nutraceuticals and antioxidant deficient community in the saline prone area around the world. However, further details experimentation is required to confirm the standardization and stabilization of functional components of vegetable amaranth for extraction of juice as drinks.

## Data Availability Statement

All the data supporting the conclusions of this article is provided within the article.

## Author Contributions

US initiated the research work, conceived the study, performed biochemical analysis, statistical analysis, drafted, edited, interpreted data, and prepared the manuscript. US, MH, and MI performed the experiments. SO edited the manuscript, provided valuable suggestions during the experiment. All authors contributed to the article and approved the submitted version.

## Conflict of Interest

The authors declare that the research was conducted in the absence of any commercial or financial relationships that could be construed as a potential conflict of interest.
